# A ride through the epigenetic landscape: aging reversal by reprogramming

**DOI:** 10.1007/s11357-021-00358-6

**Published:** 2021-04-06

**Authors:** Lucas Paulo de Lima Camillo, Robert B. A. Quinlan

**Affiliations:** 1 Department of Chemistry, Brown University, Providence, RI USA; 2 Nuffield Department of Medicine, University of Oxford, Oxford, UK

**Keywords:** Epigenetics, Aging, Reprogramming, Rejuvenation

## Abstract

Aging has become one of the fastest-growing research topics in biology. However, exactly how the aging process occurs remains unknown. Epigenetics plays a significant role, and several epigenetic interventions can modulate lifespan. This review will explore the interplay between epigenetics and aging, and how epigenetic reprogramming can be harnessed for age reversal. In vivo partial reprogramming holds great promise as a possible therapy, but several limitations remain. Rejuvenation by reprogramming is a young but rapidly expanding subfield in the biology of aging.

## Introduction

For decades, aging has been the single best predictor of human mortality in developed countries [1]. It is the major risk factor for several of the top causes of death, such as cardiovascular disease and cancer [2–4]. Organismal aging also greatly enhances the susceptibility to chronic diseases, such as diabetes, neurodegeneration, and metabolic syndromes [5]. As a complex, multi-factorial biological process, it has been typically defined by the presence of specific hallmarks [6]. Those include loss of proteostasis, mitochondrial dysfunction, genomic instability, and epigenetic alterations. Nevertheless, multiple theories have been proposed to explain the mechanism behind the convoluted aging process, many relying on only one factor. The “cross-linkage theory of aging” explains the loss of proteostasis as being due to increased hazardous crosslinking between cellular proteins [7]. The “free radical theory of aging” posited that elevated reactive oxygen species (ROS) and the resultant accumulation of cellular damage are responsible for the aging phenotype [8, 9]. Somatic DNA damage and epigenetic modifications have also been at the core of other aging theories. The “information theory of aging,” proposed by David Sinclair in 2019, suggests that loss of epigenetic information through time, like a scratched vinyl disc, is the basis for age-associated cellular deterioration [10]. Even though no theory has been proved beyond doubt, mounting evidence indicates that specific modifications in epigenetic marks are responsible for cellular and organismal aging.

According to the NIH Epigenomics Roadmap Project, “epigenetics refers to both heritable changes in gene activity and expression (in the progeny of cells or individuals) and also stable, long-term alterations in the transcriptional potential of a cell that are not necessarily heritable” [11]. Changes in histone variants, histone post-translational modifications (PTMs), DNA methylation (DNAm), among others affect gene expression and packing of chromatin, the DNA-protein complex, and fall under the field of epigenetics. These alterations are mediated by several enzymes that act as readers and modifiers, particularly histone methyltransferases (HMTs), demethylases (HDMTs), acetyltransferases (HATs), and deacetylases (HDACs). The cellular epigenetic state is a dynamic interplay of all these components and changes over time and with environmental stimuli.

Isogenic studies across species demonstrate the contributory role of epigenetics in aging. In budding yeast (*Saccharomyces cerevisiae*), early stochastic epigenetic changes markedly determine single-cell replicative lifespan [12]. Worker and queen bees possess the same genetic information, yet display strikingly different phenotypes and lifespan [13, 14]. In mice, a precocious aging phenotype can be induced by chemicals that disrupt epigenetic marks during development [15]. In humans, a similar divergence is observed with twin studies. Herskind et al. estimated that only about 25% of the variance in longevity could be attributed to the identical genetic makeup of monozygotic twins [16]. Epigenetic marks early in life are virtually identical, whereas they differ later on through so-called epigenetic drift [17]. Divergent epigenetic signatures can thus explain phenotypic differences between isogenic twins [18]. This epigenetic drift can be observed early in yeast. After an initial stochastic period, one of two epigenetic aging routes is committed to under the same genetic background and environment, resulting in a 50% replicative lifespan difference [12]. Overall, epigenetic variations are ubiquitous regulators of the aging process in various of organisms across several kingdoms.

The irreversibility of aging was assumed as recently as the end of the twentieth century [19], partly because DNA double-strand breaks and mutations, thought to be one of the causes of aging, accumulate with time [20–24]. However, genetic damage is not always correlated with aging [25]. Around that time, epigenetic modifiers such as yeast Sir2 were known to be implicated in aging, and their overexpression extended lifespan [26]. Most interventions, such as calorie restriction, slowed aging. With the discovery that aged, differentiated cells can be reversed to phenotypically young, embryonic-like stem cells [27], developmental reversal was shown to be attainable. This procedure is referred to as “epigenetic reprogramming.” Recent studies have begun to explore its use in inducing age reversal by modifying the epigenome [28, 29]. Nowadays, it is known that aging can be slowed, paused [30, 31], and even reversed.

**Fig. 1:**
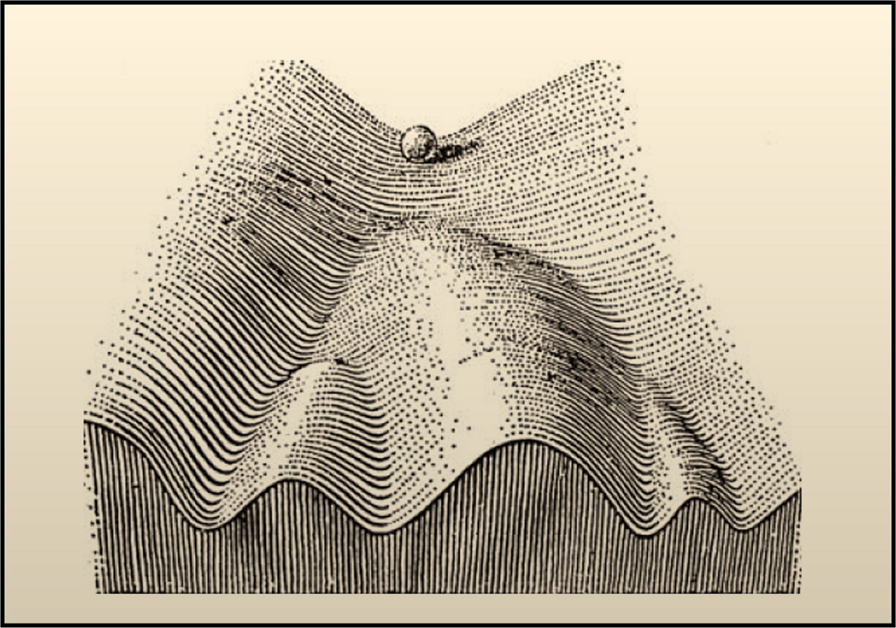
Drawing of the original epigenetic landscape proposed by Waddignton. Figure from [32]

**Fig. 2:**
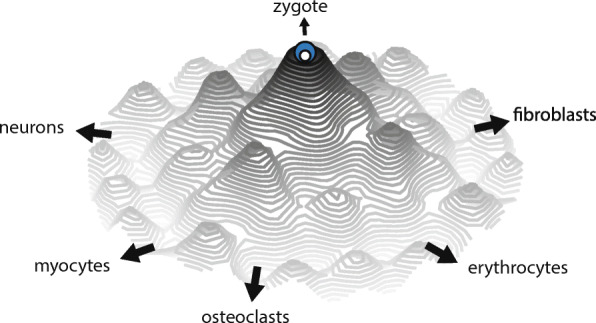
Schematic of the malleable epigenetic landscape, with height denoting epigenetic instability and each location, a specific epigenetic state

Recurrent imagery often used in developmental biology is the “epigenetic landscape” (Fig. 1 [32]), which facilitates the understanding of the aging process. After coining the term epigenetics as the “science concerned with the causal analysis of development,” Waddington created the landscape analogy in the middle of the twentieth century [32, 33]. It originally served to represent the initial stochasticity and later determinism of cell differentiation in organismal development with a marble rolling down a landscape with several valleys. The marble faces several branching points on the landscape, i.e., the choices for cell fate determination. The original epigenetic landscape is useful to visualize simple developmental pathways but limited since the topography is static. Hence, throughout this article, a modified landscape based on the malleable free-energy diagram will be used to explore the epigenetic modifications associated with aging and epigenetic reprogramming (Fig. 2). There are several local maxima and minima, with the height representing epigenetic instability and the location, a single epigenetic state. Going down the landscape can be imagined as losing epigenetic plasticity potential.

Here, this article will briefly review the findings of age-related epigenetic changes from recent studies (for more information, see Kane et al. [34]). Then, epigenetic modifications driven by reprogramming will be discussed, alongside the potential to revert aging and its limitations. Finally, reprogramming will be explored alongside other well-established life-extension interventions.

## Sliding down the epigenetic landscape: age-related epigenetic modifications

During mammalian development, the zygote is the first step in a series of preprogrammed events that result in a fully fledged adult. This single cell lies at the epigenetic landscape’s central and highest peak (Fig. 2). As time progresses, the zygote-marble descends the mountain through a succession of ever deeper valleys, ensuring cell fate stability. As the marble continues along its trajectory, several epigenetic modifications occur as it distances the center. Those include chromatin remodeling, differential histone PTMs, accumulation of histone variants, and regional hyper and hypo DNAm [3, 35].

### Reduced global heterochromatin

A gradual, net loss of heterochromatin with advancing age, observed across organisms from yeast to mammals, has been the focus of the “heterochromatin loss model of aging” [36]. It proposes that a reduction in densely packed DNA is the culprit for age-related phenotypes. The chromatin’s elemental structural unit is the nucleosome, an octamer composed of histone proteins (canonically H2A, H2B, H3, and H4) with DNA wrapped around [37, 38]. It is the basis of a significant portion of epigenetic regulation.

Supranucleosomal chromatin organization plays a key role in gene expression [39]. Transcription is affected by distinct DNA accessibility due to specific local crowding conditions in the DNA microenvironment. Loss of heterochromatin has been associated with global transcription increase [40], but packing-density also controls the genomic information space, being positively correlated with intercellular transcriptional heterogeneity [39, 41]. Elevated chromatin scaling is characteristic of cancer cells [41]. These modelling studies highlight the importance of maintaining appropriate genome topology since several diseases, including aging, are connected to reduced heterochromatin [42, 43]. Increased differential transcription steadily appears in eukaryotes with aging, from yeast to humans [36, 44–47]. In fact, the anti-inflammatory drug Celecoxib, also an adjuvant that modifies chromatin scaling [39], has been shown to extend lifespan in C. *elegans* [48]. The study presents evidence that the extension was achieved through PDK-1 activity modulation, but another explanation is a differential expression of the protein based on macrogenomic chromatin regulation.

Several molecular events are responsible for less dense genome topology. Across the genome, H1 linker histones compact chromatin by binding to short DNA segments in between nucleosomes, effectively folding the DNA-protein complex [49]. Reversible phosphorylation of serine and threonine residues at the C-terminal tail of H1 histones are responsible for regulating H1 packing behavior [50]. Individuals with a deletion encompassing these residues in one of the multiple copies of the gene display phenotypic attributes of premature aging [50]. Overall, their fibroblasts show more nucleoid relaxation, less condensed chromosomes, and higher nucleolar instability than controls [50]. Loss of silencing of nucleolar ribosomal DNA (rDNA) is also known to promote aging in budding yeast [26, 51, 52]. It is partly caused by core histone protein reduction since roughly half is lost in yeast replicative aging [53, 54]. A similar decline has been detected in the worm *Caenorhabditis elegans* [55], fruit fly *Drosophila melanogaster* [46], human fibroblasts [56], and senescent human cells [57]. Overexpression of histone proteins increases chromatin compaction and organismal lifespan across species [54, 58]. Paradoxically, histone transcripts increase with aging due to heterochromatin reduction, but functional protein synthesis is further reduced, leading to a net loss of nucleosome occupancy [40]. The consequences range from DNA damage and chromosomal translocations to integration of hazardous nucleic acids into the nuclear genome, such as mitochondrial DNA (mtDNA) and transposons [40]. Nevertheless, more open chromatin is not always detrimental. A slight reduction of 15% in histones H2A and H2B expression decreases chromatin packing but extends replicative lifespan in yeast [59]. A cellular response mediates such alterations through TOR, an important nutrient-sensing pathway that regulates aging. Even though chromatin density is an important epigenetic factor, numerous other epigenetic factors are correspondingly influential during organismal aging.

### Histone post-translational modifications

A wide range of chemical alterations occurs on histones, most notably acetylation and methylation [60]. Over 1000 different modifications can occur in the histone tails and histone globular domains [61]. This network of histone changes is highly complex and mediates the precise regulation of gene expression. Some factors that affect the distinct behavior of the modifications include the histone residue’s location, the histone’s gene position, and the composite contribution of multiple alterations.

In general, histone acetylation has been thought to facilitate gene expression and be more prevalent during aging because of elevated global transcription. Levels of histone H4 lysine 16 acetylation (H4K16ac) increase with aging in yeast and interventions that lower its abundance increase lifespan [53]. For instance, deletion of a component of the H4 HAT NuA4 promotes replicative longevity in yeast [62]. Deleting SAS2, another HAT that acetylates H4K16, extends yeast replicative lifespan [53]. Sir2, an HDAC known to promote longevity, deacetylates H4K16Ac and increases yeast lifespan [53]. As another example, H3K27ac is elevated in aged human skeletal muscle [63]. Targeting the translation of HAT p300 with short hairpin RNA extends replicative lifespan in human fibroblasts; overexpression of p300 shortens it [64]. However, hypoacetylation is not always beneficial. Loss of function of the H3 deacetylase complex Rpd3 delays aging in yeast [65] and in the fruit fly [66]. Moreover, global loss of H3K27ac is observed during aging in human and mouse brains, and in human hematopoietic cells [67, 68]. Age-upregulated genes lose H3K27ac at both promoters and gene bodies, whereas in age-downregulated genes only in the promoter, suggesting a suppressive effect of gene-body H3K27ac [67]. Sodium butyrate and suberanilohydroxamic acid, HDAC inhibitors that increase global H3K27ac, downregulated age-upregulated genes and upregulated age-downregulated genes, restoring homeostasis in the mouse brain. For some modifications, a precise level of histone acetylation is necessary for optimal longevity. For instance, H3K56Ac levels reduce with aging in yeast but increasing or decreasing H3K56Ac by deleting HATs (Hst3, Hst4) and HDACs (Rtt109) shortens lifespan and disrupts genomic stability in yeast [53, 54, 69]. It is worth highlighting how some of the age-related histone changes are species- and cell-type-dependent.

Histone methylation can cause both gene activation and silencing, with the former typically increasing and the latter decreasing with age [44]. Reduction of global H3K4 trimethylation (H3K4me3) marks, an indicator of transcriptional activation, through knockdown of HMTs prolongs lifespan, whereas knockdown of H3K4me3 HDMTs accelerates aging in C. *elegans* [70]. In old flies, H3K4me3 levels are altered [46]. On the other hand, silencing histone methylation marks, such as H3K36me3, H3K27me3, and H3K9me3, generally leads to age-related decline in organisms. H3K36me3 levels lower due to replicative aging in yeast [71] and H3K27me3 is depleted with aging in C. *elegans* and human cells [72]. An abrupt surge in the levels of H3K27me3 demethylase UTX-1 is highly associated with mortality in C. *elegans* [72]. Decreased H3K9me3 is observed in old flies compared to young flies [46], and a similar reduction occurs in mouse and human bone marrow stromal cells [73]. However, in fly heads specifically, the opposite is true [74]. In fact, a progeroid mouse model displayed increased levels of H3K9me3 and defective DNA repair within dense chromatin, and lowering H3K9me3 levels partly reversed the precocious aging phenotype [75]. The above studies highlight how different cell types display distinct age-related epigenetic modifications. Similarly to Cheng et al. [67], perhaps the location of the histone PTM within a gene might determine activating or repressive behavior that could explain the tissue variations. Further studies are necessary to elucidate the role of histone methylation marks in various positions across the genome.

Several other poorly understood histone marks display altered levels with aging. Formylation is the second most abundant histone lysine acylation in mice livers, only behind acetylation [76]. It more than doubles with aging [76]. Aliphatic acylations and advanced glycation end products (AGEs) in general increase by approximately 50%. AGEs disrupt chromatin organization, but the role of each modification in aging has not been well-documented [77]. Permanent oxidative stress markers skyrocket, particularly with the oxidation of methionine sulfoxide to methionine sulfone increasing by 5–10 fold [76]. Citrullination, believed to be involved in DNA repair, increases by approximately 50% [76]. Further research is required to understand the composite role of all these different histone modifications in gene regulation. Accumulated DNA damage might drive some of these changes [78], but it is unknown how histone PTM epigenetic information is lost.

### Chromatin modifier changes

Highly sophisticated interactions among histone proteins, nucleosome remodeling complexes (NRCs), histone modifiers (methylases, acetylases, etc.), and transcription factors alter during aging. Variants of the canonical histone proteins regulate chromatin dynamics, from assisting packing to DNA repair [79, 80]. Histone variant H3.3, initially thought to have no functional significance, accumulates in mouse brains over time [81, 82] and appears to drive cellular senescence [83]. MacroH2A, a variant associated with both transcriptional activation [84] and repression [85], increases in mice, primates, and human fibroblasts with aging [86]. Nucleosome remodeling complexes also play a role. Nucleosome remodeling deacetylase (NuRD) malfunctions in aging [87]. Isw2 and Chd1, ATP-dependent NRCs, are detrimental to yeast replicative lifespan [62]. RNA interference of the NRC SWI/SNF abolishes longevity extension in some cases in *C. elegans* [88]. Chromatin modifiers, such as HATs, HDACs, HMTs, and HDMTs, also modulate aging [53, 54, 62, 64–66, 72]. Another component is a change in transcription factors, which play key roles in DNA accessibility and modification [89, 90]. For example, loss of the transcription factor Slug in mice causes an aged phenotype in vivo [91].

### DNA methylation

Several dynamic modifications are present in the DNA of most eukaryotes and are relevant to aging. Among them, 5’-cytosine methylation (5mC) is the most frequent, typically occurring at locations of a cytosine followed by a guanine (CpG sites) [92]. Other unfamiliar cytosine additions also exist, such as hydroxymethylation (hmC), formylation (fC), carboxylation (caC), and 4’ methylation [93, 94]. Even 6’-adenine methylation has been observed [92, 94, 95]. 5mC has been assumed to dampen gene expression through steric hindrance of transcription factors, but it might be involved in nuanced raised expression depending on the position in the genome [96–101]. For the most part, 5mC methylation dwindles during mammalian aging [102–108], although some studies using modern techniques do not corroborate such global findings [109, 110]. Some specific, apparently important CpG sites are, in contrast, hypermethylated [111–114]. During development and aging, there is a methylation peak, since embryonic stem cells and old cells are hypomethylated. Accordingly, a surge in CpG 5mC has been shown in infants from 6 to 52 weeks of age [100]. Most importantly, age-related 5mC hyper- and hypomethylation is localized at particular genomic loci [111, 115, 116].

This differential hyper- and hypomethylation across the genome can be used to accurately predict age and mortality [101, 111, 117–119]. Machine learning methods allied with CpG epigenetic data were harnessed to create the so-called epigenetic clocks (see Horvath et al [120]). 353 CpG sites in Horvath’s clock and 71 in Hannum’s clock precisely calculate a person’s age with a median error of less than four years [101, 111]. Horvath’s clock is so accurate that embryonic stem cells, which are only present before birth, possess slightly negative age [111, 121]. Even the epigenetic drift can be quantified given the higher variance of DNAm age later in life [101]. DNA methylation is intrinsically related to aging, and its genome pattern is universal across eukaryotes [94]. Even then, age-related differential DNA methylation is not solely responsible for the aging phenotype. Some species such as the fruit fly virtually lack 5mC [122].

### Non-coding RNAs

Non-coding RNAs (ncRNAs), long believed to arise from transcriptional errors, are key players in epigenetic regulation [123]. They finely modulate messenger RNA (mRNA) transcription, splicing, and degradation [124] and assist in the maintenance of proper genome topology [125]. They are ubiquitous in the human transcriptome. More than half of the human genome is transcribed [126, 127], giving rise to a multitude of transcripts. ncRNAs are both positively and negatively correlated with aging. Micro RNAs (miRNAs), a class of small ncRNAs, are generally downregulated in old compared to young eukaryotes [128–130], but some delay or accelerate the aging phenotype across species [52, 131–134]. Long ncRNAs can also be detrimental or beneficial for the aging phenotype. High Gas5 expression, a type of long ncRNA, is related to impaired learning in mice [135]. At the same time, overexpression of Sarrah, another ncRNA, improves cardiac function in mice [136]. Overall, increased transcription of most long ncRNAs is damaging because of elevated R-loop formation, a three-stranded nucleic acid structure [69, 137]. It is more prone to DNA damage and leads to cellular senescence. Some long ncRNAs can even form a DNA-DNA-RNA triple helix [136]. The role of ncRNAs in aging is becoming clearer with recent studies, but their role in epigenetic modulation is certainly crucial for the aging process.

### Transposition

The “transposon theory of aging” posits that transposable elements (TEs), dubbed “jumping genes” for their excision and reintegration potentials, cause cellular degeneration and aging [138]. They are usually silenced during youth, but as heterochromatin is lost, they become activated. Chromatin packing deregulation directly elicits expression of transposable elements with age in fruit flies [58] and mice [139]. The transcript levels increase as the cellular mechanisms that suppress integration become insufficient to prevent it. Overexpression of genes that stabilize heterochromatin and lamivudine (a drug that targets the TE machinery component reverse transcriptase) restrains TEs and extends lifespan [58]. As expected, specific TEs are both silenced and expressed differentially [140]. Almost all have biased de novo insertions in the genome, but long interspaced repeat element 1 (LINE1), the most abundant human TE [141], is not generally affected by the presence of heterochromatin [142]. LINE1 activation contributes to age-related inflammation and cellular senescence [139]. Stavudine, a LINE1 reverse-transcriptase inhibitor, rescues the young inflammation profile in mice [143] and lamivudine partly inhibits the cellular senescence phenotype [139]. The stavudine-treated group had approximately a 30% lower DNAm age [143]. These results suggest that transposition is not merely correlated with age but one of the causes of aging.

## Climbing back up the epigenetic landscape: reversing epigenetic modifications by reprogramming

**Fig. 3:**
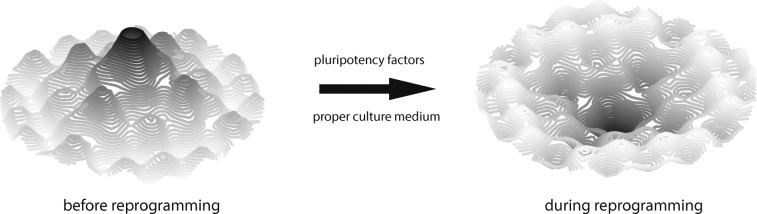
Transition of the epigenetic landscape during reprogramming. The peaks become grooves, pulling the marble towards the center

The first notable experiment that showed that a differentiated somatic cell still contains all the necessary genetic information to produce an entirely new organism was somatic cell nuclear transfer (SCNT) [144]. The process of completely modifying a cell phenotype was named reprogramming. More recently, in 2006, it was shown that exogenous expression of the four factors Oct3/4, Sox2, Klf4, and c-Myc (OSKM) is sufficient to transform fibroblasts into induced pluripotent stem cells (iPSCs) [27]. Forced expression of the pluripotency factors modifies the landscape by effectively flipping the topography (Fig. 3). The central peak where the marble initially dwelled becomes an abyss. It pulls the marble towards the center again to reduce epigenetic instability, representing the loss of somatic identity and gain of pluripotency.

In order for reprogramming to work correctly, efficient activation of the pluripotency network is the only requirement. Although the original OSKM combination is still used today, the complete cocktail of factors is not essential. Earlier, removing any of the factors did not elicit reprogramming [27]. With advances in the culture medium, three out of the four was enough, albeit with a lower efficiency [145]. SK alone could reprogram highly proliferative differentiated cells [145]. With the use of the clustered regularly interspaced short palindromic repeats (CRISPR) system, sole Oct4 or Sox2 overexpression lead to activation of the pluripotency circuitry [146]. There is an initial stochastic phase followed by a hierarchical, deterministic activation of certain genes, with Sox2 appearing to be a central node [147]. Any combination of factors that induces the network and produces the correct levels of pluripotency proteins can successfully reprogram [27, 148, 149].

As will be explored below, several epigenetic marks are effectively reversed during reprogramming. This remarkable process is being studied as a potential therapy against aging. Nevertheless, it has several limitations. Low efficiency, formation of teratomas (an extremely aggressive form of cancer), and persistence of certain epigenetic marks are some of the barriers to be overcome.

### Epigenetic changes during reprogramming

Given the high degree of similarity between embryonic stem cells (ESCs) and iPSCs [27, 150–152], it is expected that some changes that occur during reprogramming might be a simple reversal of age-related epigenetic modifications. A few alterations, such as global hypomethylation and heterochromatin loss, get exacerbated during reprogramming, whereas others, such as telomere attrition, are rewound.

A recent longitudinal analysis of the transcriptome of the reprogramming intermediates of mouse embryonic fibroblasts (MEFs) identified key steps in the process [153]. Principal-component analysis highlighted five stages. 5mC DNA methylation declined markedly in the first step and then steadily dropped by half until the final stage when, intriguingly, it increased, but not enough to reach pre-reprogramming levels. These results suggest a first wave of demethylation to erase the differentiated cell program that primarily targets promoters, enhancers, and upstream regulatory elements. It is accompanied by a second, more gradual demethylation wave, which mainly affects gene bodies. Finally, a rapid surge in methylation, which may surpass progenitor-level global methylation [154], most likely determines the pluripotency program in the last step. The most important demethylation targets are pluripotency genes and their respective regulatory regions [27, 155]. Some types of transposons are demethylated, such as LINEs, others remain methylated, such as intracisternal A particle elements, and active retroviruses are silenced [145, 153, 156]. All these methylation changes contribute to the slightly negative age of iPSCs in Horvath’s epigenetic clock [111, 121].

When it comes to 5hmC, there is a global increase when comparing iPSCs to human fibroblasts [157]. TET1, the main enzyme responsible for regulating hydroxymethylation, is activated early during reprogramming [153] and is present in higher levels in iPSCs [157]. It is required for proper erasure of the previous cellular program during reprogramming [153]. Roughly 85% of the differentially hydroxymethylated regions are hyper hydroxymethylated, mostly concentrated near telomeres [157]. Gene expression is inversely correlated to 5hmC in transcriptional start sites [157]. A consequence is both hypomethylation and hypo hydroxymethylation in the regulatory elements of pluripotency factors [157]. Nevertheless, 5mhC is not a mere consequence of the oxidation of previously 5mC DNA, as increased hydroxymethylation is found in regions of both hyper and hypomethylation [157]. These observations suggest that 5hmC plays a role in regulating the pluripotency circuitry.

Another age-related epigenetic change that becomes exacerbated in reprogramming is the loss of heterochromatin. In OSKM reprogramming, OSK are the pioneer factors to induce global opening of chromatin [147]. Such chromatin unpacking has been observed even with in vivo reprogramming in mice, as DAPI, a DNA staining compound, elicits a weaker, more delocalized signal [158]. Heterochromatin protein 1*β* (HP1*β*), involved in gene expression regulation and DNA repair, is much more mobile in iPSCs than human fibroblasts [159]. HP1*β* mobility can even be used as a proxy to track reprogramming progress [159]. Recent advances in 5C and high-throughput sequencing have allowed a finer look at genome architecture modifications [151]. Topologically associating domains (TADs) show striking differences before and after reprogramming [151, 160]. Sliding back down to the center of the epigenetic landscape involves breaking and reforming cell-type-specific patterns of 3D interactions, albeit not all of them [151]. The more iPSCs are passaged, the more they acquire ESCs-specific higher-order chromatin connectivity [160]. However, a finer resolution in TAD mapping shows that iPSCs subdomain connectivity is not completely ESC-like [151], even though bigger domains become virtually indistinguishable.

There are also several changes observed with histone proteins and histone PTMs. In the Oct4 and Nanog promoters, H3K9 methylation remained constant, but H3 acetylation was much higher, indicating activation of pluripotency factors [27]. H3K9me3 and H4K20me3, silencing marks, decline globally but particularly at pericentric repeats and telomeres [156, 158]. H3K4me3 and H3K27me3, activating and repressive marks, respectively, change in two waves [155]. Initially, H3K4me3 relocates to other regions and H3K27me3 increases globally, particularly in differentiated cell-type-specific genes. Later, H3K4me3 levels surge and H3K27me3 moves to other areas [155]. These changes are concomitant to the first 5mC demethylation wave and the second 5mC methylation increase, indicating the activation and suppression of different genes across time in reprogramming [153]. In addition to histone PTMs, all the canonical histones are upregulated [161]. Even histone variants are likely to be repositioned through nucleosome remodeling, as H3.3 shifts downstream from promoters [162, 163].

Reprogramming does not always completely erase the previous cellular program, with some epigenetic remnants maintained after the process. The first induction of iPSCs already demonstrated that epigenetic marks, such as 5mC DNA methylation and H3K9me3, are not fully reversed on the promoters of pluripotency genes to levels seen in ESCs [27]. Moreover, genome-wide hydroxymethylation analysis of iPSCs derived from human fibroblasts highlights the presence of 20 large-scale regions with enduring 5hmC [157]. Tissue-specific residual 5mC methylation remains in iPSCs and these methylation foci might interfere with differentiation into some cell types [154, 164]. Moreover, some age-related methylation marks remain in iPSCs derived from aged donors. Based on Horvath’s epigenetic clock, there is a correlation, although weak, of the progenitor cells’ age and the epigenetic age of iPSCs [121]. Reprogramming of old cells result in a 5% global methylation increase [121]. These studies suggest iPSCs are primed to return to their original state more easily, as the trail traced on the epigenetic landscape is always a two-way pathway. Nevertheless, this epigenetic memory in reprogramming is generally assumed to be insignificant [165].

Overall, reprogramming takes a different route from a simple sequential reversal of age-related epigenetic changes. For instance, DNA methylation and global heterochromatin, which peak during youth, do not pass through the same peak. Another detour is evidenced by fibroblasts reprogrammed with OSKM taking a longer route if Oct4 is present, potentially reacquiring fibroblast features [145]. Oct4, in this case, leads to a loss of imprinting, misregulation of polycomb targets, and epigenetic anomalies, meandering through valleys on the epigenetic landscape. Different pathways to the center of the landscape can be taken with distinct epigenetic changes, as local minima become local maxima and vice versa. For more information on epigenetic changes during aging, see review by Papp and Plath [166].

### Partial epigenetic reprogramming

In order for epigenetic reprogramming to be harnessed for the treatment of aging, dedifferentiation must not occur. The marble cannot return to the center, as complete in vivo reprogramming is a dangerous process that might lead to severe health problems or death. Hence, partial reprogramming, i.e., reversing age-related epigenetic changes without pluripotency acquisition, is the only feasible use of this technique. It has already been shown that reprogramming cells to increase “stemness” without reaching pluripotency drastically reduces the aged phenotype [167]. There is a steady, dramatic decrease in DNAm age during reprogramming [168]. The literature on this topic is current but scant and is summarized in Table 1.

**Table 1:** Studies of partial epigenetic reprogramming

Model	Reprogramming method	Length of induction	Results	Duration of effects	References
Mouse progeroid model (LAKI 4F mice)	in vivo and in vitro inducible OSKM	Cyclic in vivo induction and transient in vitro induction	Restored H3K9me3 and H4K20me3, alleviated aging phenotypes, and increased lifespan.	Aged-phenotype slowly reacquired after 4 and 8 days of withdrawal in vitro	[28]
Aged human fibroblasts and endothelial cells	in vitro OSKMLN mRNA tranfection	4 days	Restored H3K9me3, Sirt1, HP1*γ*, and decreased *β*-galactosidase and DNAm age.	Most changes endured for at least 6 days	[29]
Senescent human fibroblasts	in vitro OSKML plasmid	Continuous expression	Restored HP1*β* mobility and decreased *β*-galactosidase.	Aged-phenotype slowly reacquired after 9 days	[159]
Mouse club cells	in vitro inducible OSKM	3 weeks	Increased proliferative capacity.	Mostly reversed phenotype after 2 weeks of withdrawal	[174]
Mouse embryonic fibroblasts	in vitro OSKML + p53 shRNA episomal plasmid	Single transfection	No rejuvenation phenotype.	No change	[172]
Middle-aged human fibroblasts	in vitro inducible OSKM	10–17 days	Restored H3K9me3, decreased DNAm age, decreased transcriptional age, restored collagen expression.	Unspecified	[171]

Sarkar et al. published an analysis of transient expression of reprogramming factors in aged human fibroblasts and endothelial cells [29]. They transfected OSKM plus Lin28 and Nanog (OSKMLN) mRNAs for four days and performed several assays two days later. Transcriptome analysis showed a clear similarity between treated and young cells without activation of the pluripotency network. Similar or higher levels of heterochromatic H3K9me3 were found, which is intriguing given that full reprogramming depletes that histone PTM [156, 158]. This same epigenetic change was seen in partial reprogramming in mice fibroblasts [28]. Protein levels of Sirt1, an enzyme that promotes longevity [169], and HP1*γ*, an isoform of HP1*β*, increased. *β*-galactosidase, a hallmark of senescence [170], and proinflammatory senescence-associated secretory phenotype decreased, as seen in [28] as well. Strikingly, even Horvath’s epigenetic clock calculated an age reduction on average of 3.4 years. These youthful restorations mostly endured for the next 4 and 6 days after the interruption, although more moderately. In mice fibroblasts, the prior phenotype slowly restored after 4 and 8 days of interruption [28].

Reik at al. recently conducted a similar experiment using human fibroblasts from patients between 38 and 53 years [171]. The researchers used lentiviral transduction to transport an OSKM cassette inducible by the antibiotic doxycycline and started the reprogramming process. After 10 to 17 days, doxycycline was removed and the morphological changes observed upon reprogramming were reversed. In the successfully partially reprogrammed cells, a median drop of about 30 years in both the transcriptional clock and Horvath’s DNAm clock was observed, a much sharper decrease than observed by Sarkar et al. [29]. The youthful phenotype, characterized as a restoration in H3K9me3 levels and increase in collagen production, remained for an unspecified amount of time. Notwithstanding such encouraging results, the fibroblasts did momentarily lose their morphology during the transient reprogramming.

Sarkar and Reik’s reports indicate great promise in partial reprogramming, but other studies reported more cautious results. It is not surprising that transient reprogramming results in transient rejuvenation. OSKML reprogramming in senescent human fibroblasts for 9 days—it takes 40 to reach pluripotency in this case—did decrease *β*-galactosidase and increased HP1*β* mobility [159]. However, at day 12, the previous senescence phenotype returned [159]. Another study analyzed reprogramming in human mesenchymal stromal cells maintained in the same cell-specific medium [172]. Compared to control cells, they entered replicative senescence simultaneously and showed the same levels of *β*-galactosidase and p16, another senescence-associated marker [173]. Transient expression might lead to cell-fate anomalies. Partial OSKM reprogramming in mature lung epithelial cells over three weeks generated a non-natural progenitor [174]. Differently from the mature lung epithelial cells, the generated cells were easily expanded but did not display pluripotency markers [174]. The novel cell type’s appearance might be explained by the passage through a local maximum on the epigenetic landscape that represents a normally inaccessible epigenetic state; with the partial reprogramming, the local maximum becomes a local minimum. These contrasting results might have occurred due to the use of different reprogramming methods, cells, culture mediums, and lengths of expression.

To date, the most startling study showing the potential of partial reprogramming in tackling aging was conducted by Ocampo et al. [28]. The researchers used a progeroid mouse line that can express OSKM in vivo when given doxycycline, in contrast to in vitro conditions of all previously mentioned studies. Two days of induction followed by five days of abstention did not cause cancer or activation of pluripotency factors besides OSKM. The treatment led to a striking lifespan extension of approximately 50%. Several age-associated phenotypes were reversed, including restoration of normal H3K9me3 and H4K20me3 levels and reduced senescence-associated *β*-galactosidase. Partial in vivo reprogramming may also confer tissue regeneration capacity not even observed in young animals. It appears that transient reprogramming greatly supports tissue regeneration following injury [28, 175], as partially reprogrammed cells can replenish tissues more faithfully [29]. If the expression of pluripotency factors is not immediate after the damage, broad regeneration does not occur [175]. Since phenotypic plasticity is correlated with greater chromatin scaling [39, 41], partial reprogramming, by briefly opening chromatin [159], promotes an epigenetic environment that is fruitful for tissue regeneration. Tissue damage stimulates phenotypic plasticity and reprogramming as well [176]. In vivo reprogramming holds great promise for age reversal and tissue regeneration.

### Limitations

Even with state-of-the-art techniques, several limitations hinder the applicability of reprogramming to confront aging. Besides the pharmacological difficulties of delivering a system capable of expressing the pluripotency factors, low efficacy, high heterogeneity, and severe side effects are pressing problems.

**Fig. 4:**
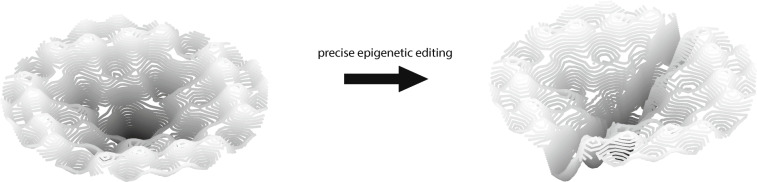
How epigenetic editing of, for instance, promoters of pluripotency genes can increase the likelihood that the marble will take a quick route towards the center by the carving of a valley

Reprogramming outcomes vary widely based on the reprogramming method, tissue source, progenitor cell age, and the cellular environment. An analysis of multiple reprogramming methods revealed drastic differences upon expression of the same set of pluripotency factors [177]. There was no methylation variation across methods, but retroviral-derived iPSCs were 13.5% aneuploid while mRNA-derived iPSCs were only 2.3%. Clustering of transcriptomes based on reprogramming techniques showed that, in general, similar procedures group together [152]. Tissue source may prime iPSCs to differentiate into their progenitor cells [154, 164]. The age of the progenitor cells also leads to heterogeneous reprogramming. There is evidence that donor age does not influence efficiency (typically 0.01–0.1%) even though it alters the number of successful cultures, leading to an overall lower yield [121, 164, 178]. Donor age also affects chromosomal abnormalities, DNA damage response, and apoptosis [179]. Culture medium affects variability and efficiency as well. Exchanging the culture medium between good and bad cultures narrows the difference in efficiency by 60% [178]. The ratio of inflammatory cytokines in the environment is partly responsible for the variability [176, 178], and might explain the increased heterogeneity in cultures of old progenitor cells. NF-*κ* B, a protein involved in cytokine production, and interferon-gamma, a cytokine, are likely reprogramming barriers [180]. Another reason for high heterogeneity in reprogramming is the differential epigenetic signature in pluripotency loci [181]. Precise placement of epigenetic modifiers where transcription is needed can greatly improve reprogramming [181, 182], as a valley is carved on the epigenetic landscape leading straight to the center (Fig. 4). Nevertheless, low efficiency and high heterogeneity can be solved with an improved cocktail of reprogramming factors and miRNAs. Efficiency of over 800% has been achieved since iPSC generation efficiency is calculated as the number of colonies generated per progenitor cells [183]. In single-cell experiments, 90.7% were successful. These results highlight the difficulty in generating reprogrammed cells, even though current protocols are close to converting 100% of the cells to iPSCs.

Full in vivo reprogramming is severely detrimental to health, leading to death or cancer development in a matter of days. Continuous expression of OSKM in mice results in death in a week caused by generalized abnormal tissue growth [28, 184, 185]. Reduced continuous expression still leads to death due to teratoma formation within weeks [184–187]. Nevertheless, the oncogenic potential of in vivo reprogramming does not likely arise from new mutations, as reprogramming does not appear significantly mutagenic [179, 188]. Shorter OSKM or OSK induction (fewer than five days) does not seem to result in cancer, even though temporary abnormal tissue growth and development is observed [185, 186]. These results suggest that a loss of cell-specific phenotype precedes cancer development. Transient OSKM or OSK expression also increases, for instance, colon cancer metastases [189]. A possible reason for the increased metastatic potential is the reshaping of the epigenetic landscape, with loss of tissue-specific transcription observed in multiple metastatic cancers [190]. Interestingly, several tumor suppressor genes function as barriers to reprogramming. p53, a protein in which mutations cause several types of cancer, decreases reprogramming efficiency [156, 176, 186, 191]. The tumor suppressor locus Ink4a/Arf codes for proteins that hinder reprogramming in vitro [192], even though it seems to be necessary for proper in vivo reprogramming [176]. Another complication of using reprogramming to tackle aging is that it triggers and is stimulated by cellular senescence, one of the hallmarks of aging [6]. In regions where in vivo reprogramming is successful, there is an increase in the number of *β*-galactosidase positive cells and the microenvironment is filled with cytokines and other senescence-associated molecules [176, 186]. Interestingly, reprogramming leads to both cancer cells, which have an epigenetic age much higher than the organism [111], and iPSCs, which are much younger, evidence indicating the flattening of the epigenetic landscape. All these results indicate that reprogramming indeed flattens and flips the epigenetic landscape, making it easier for the marble to take other pathways, including senescence and tumorigenesis. It is difficult—if not impossible—to control the boundaries precisely, as tissue type, age, and the cellular microenvironment affect reprogramming. It is worth noting, however, that a reduction in senescent cells was seen with cyclic partial reprogramming [28].

**Fig. 5:**
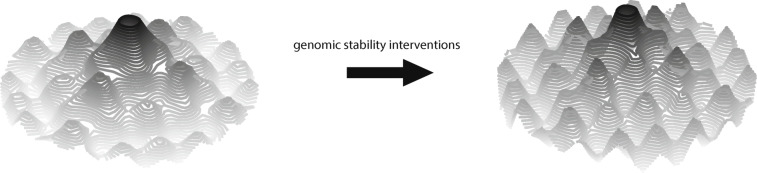
Interventions that improve genomic stability create deeper valleys on the epigenetic landscape, making it harder to change the epigenetic state

**Fig. 6:**
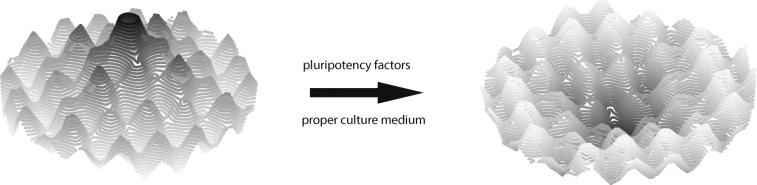
Interventions that improve genomic stability also hinder reprogramming by enhancing the barriers to reach the center in comparison to Fig. 3

Another limitation in reprogramming to tackle aging is what can be described as the age-reversal-age-extension (Arae) paradox. In the epigenetic landscape, several lifespan-extending interventions that promote genomic stability make the valleys deeper. However, reprogramming flattens the landscape so that the marble can move back towards the center, making them intrinsically incompatible (Figs. 5 and 6). It has been shown that heterochromatin pathways act as barriers to reprogramming [193, 194]. The naked mole-rat (NMR) is an extremely long-lived murine, mainly because of its remarkable genomic stability. Reprogramming of NMR fibroblasts requires a special culture medium that quite easily reprograms mouse fibroblasts [195]. Three of the most well-known longevity pathways are AMPK, mTOR, and SIRT1. Metformin, thought to be an AMPK activator, increases lifespan across species [196–199]. In human cells, a therapeutic concentration of 100 *μ* M [200] was sufficient to alleviate several aging markers [201]. Only 10 *μ* M already significantly reduced reprogramming efficiency, and this inhibition increased with concentration [202]. Rapamycin, an inhibitor of mTOR, slows aging [203–205]. The drug appears to increase reprogramming potential at low doses with a peak at 0.3 nM [206], but decrease from 1 to 20 nM in a dose-dependent manner [207]. Human physiological concentrations range from 5 to 30 nM [208] and approximately 50 nM was used in studies showing lifespan extension [209, 210]. Resveratrol, involved in the SIRT1 pathway, ameliorates several age-associated phenotypes [211]. Even though it enhances reprogramming at 0.2–5 *μ* M, the effect wanes at 10 *μ* M and efficiency is drastically reduced at 20 *μ* M [212, 213]. Nevertheless, concentrations of even 500 *μ* M extended lifespan in yeast [214], and most of the health benefits of resveratrol are conferred in the 10–25 *μ* M range in vitro [215]. On the other hand, nicotinamide, a SIRT1 inhibitor, promotes reprogramming at the same concentration it reduces yeast replicative lifespan [191, 216]. The general enhancement of reprogramming efficiency at low doses of longevity-promoting compounds might be driven by increased epigenetic remodeling, since all the aforementioned drugs affect some epigenetic modifications. However, at higher doses that more optimally extend lifespan, the elevated genomic stability might hinder reprogramming. In summary, the essence of the Arae paradox is that several interventions that slow aging can be barriers to reprogramming.

## Concluding remarks

Epigenetics and aging are intrinsically related. Epigenetic modifications predictably occur throughout development and aging. Reduced global heterochromatin, altered histone PTMs, differential DNA methylation, and others account for the bulk of age-related changes. Targeting any one of these changes ameliorates aging hallmarks and extends lifespan. Epigenetic reprogramming reverses most if not all of the age-related epigenetic modifications. Harnessing partial reprogramming seems the most promising therapy to treat aging.

Even though the bulk of research concerning reprogramming only occurred in the last decade, several major strides have been made. Nowadays, we know how physical cues may assist in the process [217]. We can avoid heightened genome instability in iPSCs derived from aged progenitors [179]. In vivo delivery of pluripotency factors is vastly improving [187], as well as tissue regeneration by reprogramming [175, 183]. Even the power of CRISPR is being used in reprogramming [146, 149, 181, 182, 218].

**Fig. 7:**
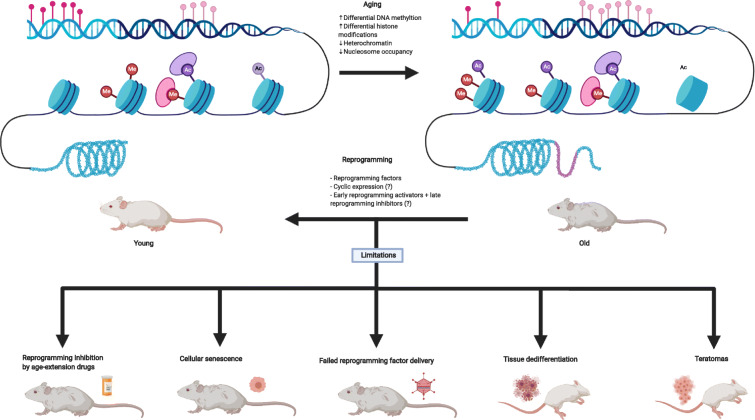
Illustration of some of the age-related epigenetic changes and the limitations and consequences of reprogramming for rejuvenation (created with BioRender.com)

There are still many limitations (Fig. 7). Whole organism delivery and homogeneous expression of pluripotency factors are challenging, currently unsolved tasks. Moreover, even though efficiency is greatly improving [183], most of the studies on reprogramming and aging still use only OSKM. Further research is also required to clarify the fine line between cancer and reprogramming. Lastly, the Arae paradox makes it unlikely that current lifespan interventions would have an additive effect alongside reprogramming.

A suggestion is an approach similar to the one shown by Ocampo et al. [28], but with the inclusion of a treatment that enhances genome stability during the absence of pluripotency factor expression. Short expression of pluripotency factors followed by administration of metformin, rapamycin, or even resveratrol would slightly flip the epigenetic landscape followed by the formation of deeper grooves. Perhaps this procedure would both improve lifespan extension and hinder the development of cancer and senescent cells. Further research is needed to check the validity of this speculative regimen.

Other combinations of reprogramming factors besides OSKM might prove more useful in decoupling age reversal from developmental reversal. It appears that the steady decline in DNAm age occurs before full reprogramming is achieved [168]. However, given the nonhomogeneous environment in vivo, there is a high risk that some cells will pass this threshold while others will likely not even experience epigenetic rejuvenation. Given the current knowledge of the pluripotency network [147], perhaps a cocktail of early reprogramming factors with late reprogramming inhibitors might reverse the aging phenotype without change of cell fate. Other well-known reprogramming barriers, such as p53, might be a barrier to full but not partial reprogramming. Further research ought to elucidate how to partially reprogram without cell dedifferentiation.

Other non-reprogramming strategies such as the use of drugs and young blood plasma are also proving promising for rejuvenation. A cocktail of metformin, human growth hormone, and dehydroepiandrosterone, rewound Horvath’s epigenetic clock by 2.5 years after one year of treatment in humans [219]. Moreover, it is known that parabiosis (the anatomical joining of two individuals) of old and young rats improves organ function in the aged animals [220]. It has recently been shown that administering young blood plasma to old rats more than halves the DNAm epigenetic age [221]. Such treatments can indirectly result in epigenetic modifications, yet are safer and less invasive than direct reprogramming. Non-reprogramming strategies might influence epigenetic marks by affecting the complex cellular regulatory networks downstream of gene expression. Because of their likely indirect effect, such treatments may not be as effective in age reversal as reprogramming; they might act as a stopgap before the challenging limitations of safe, effective in vivo reprogramming are resolved.

Aging reversal by epigenetic reprogramming is a new field of research, and not much has been studied. Nevertheless, even with all the current limitations, the future of reprogramming holds promise for the treatment of aging.
